# Treatment of Acute Kidney Injury: A Review of Current Approaches and Emerging Innovations

**DOI:** 10.3390/jcm13092455

**Published:** 2024-04-23

**Authors:** Christina Tamargo, Mohamad Hanouneh, C. Elena Cervantes

**Affiliations:** 1 Department of Medicine, Division of Nephrology, Johns Hopkins University School of Medicine, Baltimore, MD 21287, USA; 2 Nephrology Center of Maryland, Baltimore, MD 21239, USA

**Keywords:** acute kidney injury, blood pressure, dialysis, fluids, kidney replacement therapy, treatment, vasopressors

## Abstract

Acute kidney injury (AKI) is a complex and life-threatening condition with multifactorial etiologies, ranging from ischemic injury to nephrotoxic exposures. Management is founded on treating the underlying cause of AKI, but supportive care—via fluid management, vasopressor therapy, kidney replacement therapy (KRT), and more—is also crucial. Blood pressure targets are often higher in AKI, and these can be achieved with fluids and vasopressors, some of which may be more kidney-protective than others. Initiation of KRT is controversial, and studies have not consistently demonstrated any benefit to early start dialysis. There are no targeted pharmacotherapies for AKI itself, but some do exist for complications of AKI; additionally, medications become a key aspect of AKI management because changes in renal function and dialysis support can lead to issues with both toxicities and underdosing. This review will cover existing literature on these and other aspects of AKI treatment. Additionally, this review aims to identify gaps and challenges and to offer recommendations for future research and clinical practice.

## 1. Introduction

Acute kidney injury (AKI) represents a spectrum of disease characterized by changes in urine output and serum creatinine concentration. In recent years, concepts of kidney injury and failure have evolved, replacing the term “acute renal failure” (ARF) with the Risk, Injury, Failure, Loss of kidney function, and End-stage kidney disease (RIFLE) criteria, and subsequently with the Kidney Disease Improving Global Outcomes (KDIGO) criteria for AKI [1].

Just as the definition of AKI has changed, so have evaluation, prevention, and management of AKI. Guidelines recommend prompt determination of the etiology of AKI to guide therapy, and this evaluation has become more robust with the use of newer tools like biomarkers that can supplement more traditional tools (history, volume assessment, response to diuretics, urinalysis, biopsy) [2]. Pharmacologic advances have paved a way for targeted AKI therapy for certain etiologies, such as terlipressin in hepatorenal syndrome [3]. Furthermore, recent research has provided new insights on factors that can be optimized in AKI, like blood pressure and volume status, and many have adopted a “goal-directed” approach to hemodynamic therapy, with additional studies underway [4]. Dialysis modalities have been updated and diversified, offering a range of options to address different patient needs when AKI progresses to the point of requiring kidney replacement therapy (KRT) [5]. Nonetheless, gaps in understanding and management of AKI remain.

This review highlights existing literature on AKI treatment. It focuses on blood pressure targets, fluid management, vasopressor therapy, indications for KRT, KRT timing, and important drug considerations in AKI. Through these topics, we aim to expose what is known about AKI management, as well as the gaps and challenges that remain.

## 2. Blood Pressure Targets in AKI

Blood pressure control is one of the most common medical problems in various healthcare settings. In contrast to the general population, in which trials like the Systolic Blood Pressure Intervention Trial (SPRINT) have led to recommendations for lower blood pressure targets (i.e., systolic blood pressure (SBP) less than 120 mmHg) [6], blood pressure targets are typically higher in AKI and depend on both patient characteristics and AKI etiology.

### 2.1. What Is the Appropriate Blood Pressure in AKI?

Most of the data on blood pressure targets in AKI come from studies on patients with sepsis, undergoing surgery, or post-cardiac arrest. These studies generally advocate for maintaining a higher mean arterial pressure (MAP) to ensure adequate kidney perfusion. Notably, guidelines such as those from the Surviving Sepsis Campaign recommend a target MAP of 65 mmHg in septic shock [7]. MAP is a large determinant of end-organ—and thereby kidney—perfusion. In healthy states, autoregulatory mechanisms maintain kidney blood flow and glomerular filtration rate (GFR) with changes in MAP, but these mechanisms may fail in extreme blood pressure ranges or during shock states [8]. Multiple studies have sought to outline blood pressure thresholds associated with AKI and related outcomes, and these thresholds are summarized in Table 1 and described below. 

The Finnish Acute Kidney Injury (FINNAKI) study, a prospective observational study of 423 patients with severe sepsis, found that a time-adjusted MAP below 73 mmHg predicted AKI [9]. In the FEDORA randomized clinical trial (RCT) of 450 patients undergoing major elective surgery, maintaining a MAP above 65 mmHg reduced AKI risk [4]. Among 789 comatose patients who had out-of-hospital cardiac arrest, a target MAP of 63 mmHg increased risk of KDIGO stage 1 AKI compared to 77 mmHg, with no impact on severe AKI [10]. In a smaller trial of 50 patients with out-of-hospital cardiac arrest, a target MAP of 72 mmHg showed no improvement in organ injury biomarkers but did show a trend toward preserved kidney function compared to those targeting a MAP of 65 mmHg [11].

Other studies also support higher MAP targets in AKI, taking into account patients’ baseline blood pressures. Autoregulation in chronic hypertension requires higher MAPs to maintain the same protective effects [8,12]. The Sepsis and Mean Arterial Pressure (SEPSISPAM) RCT evaluated 776 patients with septic shock and found lower AKI risk with a target MAP of 80–85 mmHg versus 65–70 mmHg in those with chronic hypertension [13]. Similarly, Intraoperative Norepinephrine to Control Arterial Pressure (INPRESS), an RCT of 298 patients undergoing major surgery, favored maintaining blood pressure near baseline for superior kidney outcomes [14]. Notably, a retrospective cohort study of noncritically ill patients with AKI showed a 2.5-fold increase in severe AKI or mortality with SBP <100 mmHg compared to 120–129 mmHg in patients on antihypertensive medications at baseline. In non-hypertensive patients, the event rate was higher with higher SBP targets, stressing the importance of baseline blood pressures [15]; MAPs were not used to guide treatment in either studies which is a limitation. Finally, a prospective study of 678 patients with chronic hypertension undergoing major gastrointestinal surgery found lower AKI incidence with a target MAP of 80–95 mmHg compared to 65–79 mmHg or 96–110 mmHg [16], but there was no normotensive comparator group.

These studies suggest higher MAP targets may be beneficial, but only to a certain extent, and emphasize the importance of individualizing care to patients based on their baseline blood pressures. However, the data are not unanimous. For example, one RCT on elective cardiac surgery under cardiopulmonary bypass (CPB) found no AKI risk difference between MAP targets of 75–85 mmHg and 50–60 mmHg. However, this study had limitations, including incomplete achievement of target MAP, use of only norepinephrine for achieving the goal, and lack of baseline blood pressure consideration [17]. A systematic review and meta-analysis of RCTs concluded that targeting a higher MAP in shock and perioperative patients may not reduce the risk of AKI compared to normotension, except in shock in patients with pre-existing hypertension [18].

In hepatorenal syndrome (HRS), clinicians often aim for higher MAPs based on studies like CONFIRM (A Multi-Center, Randomized, Placebo Controlled, Double-Blind Study to Confirm Efficacy and Safety of Terlipressin in Subjects With Hepatorenal Syndrome Type 1), which showed benefit of terlipressin, a splanchnic and systemic vasoconstrictor, over placebo in improving kidney function [3]. Before terlipressin, the combination of octreotide, midodrine, and albumin or norepinephrine was used for similar mechanistic reasons—with studies titrating vasoconstrictive therapies to a MAP goal—and also demonstrated improvement in kidney function [19,20].

Another group of interest is noncritically ill patients with AKI. Although limited, a retrospective cohort study of over 1500 hospitalized patients revealed a U-shaped curve between average SBP and severe AKI or mortality, with the lowest event rate observed at SBP of 110–129 mmHg [15]. In addition to not reporting MAP, however, a major limitation to this study is that AKI etiology was not explored, highlighting a challenge in AKI adjudication, as studies indicate poor agreement among experts [21].

In summary, MAP goals in AKI are often higher than normal, but specific targets should be individualized based on a patient’s baseline blood pressure and comorbidities as well as the etiology of AKI. This is particularly relevant in individuals with pre-existing hypertension.

**Table 1:** Highlighted studies investigating blood pressure targets in acute kidney injury.

Study	Type of Study	Population	Findings
Finnish Acute Kidney Injury (FINNAKI), 2013 [9]	Prospective observational	423 patients with severe sepsis	Time-adjusted MAP **below 73 mmHg** predicted AKI
Sepsis and Mean Arterial Pressure (SEPSISPAM), 2014 [13]	RCT	776 patients with septic shock	Patients with chronic hypertension with target MAP of **80–85 mmHg** had less AKI risk compared to **65–70 mmHg**
Optimal blood pressure decreases acute kidney injury after gastrointestinal surgery in elderly hypertensive patients, 2017 [16]	Prospective randomized	678 patients with chronic hypertension undergoing major gastrointestinal surgery	Patients with target MAP of **80–95 mmHg** had less AKI compared to **65–79 or 96–110 mmHg**
FEDORA, 2018 [4]	RCT	450 patients undergoing major elective surgery	Maintaining a MAP **above 65 mmHg** reduced AKI risk
Optimal systolic blood pressure in noncritically ill patients with acute kidney injury, 2019 [15]	Retrospective cohort	1612 hospitalized, noncritically ill patients with AKI	Patients with SBP * **110–129 mmHg** had less severe AKI or 90-day mortality compared to **≤110 or ≥130 mmHg**
Substudy of Blood Pressure and Oxygenation Targets After OHCA (BOX), 2023 [10]	RCT substudy	789 comatose patients who had OHCA with presumed cardiac cause and sustained ROSC	Patients with target MAP of **63 mmHg** had increased risk of stage 1 AKI compared to **77 mmHg**

Green indicates that higher blood pressures were associated with better outcomes, and no color indicates that middle-range blood pressures were associated with better outcomes. No studies found better outcomes with lower blood pressures or blood pressure targets. Only studies that identified numeric thresholds are listed here. AKI, acute kidney injury; MAP, mean arterial pressure; OHCA, out-of-hospital cardiac arrest; RCT, randomized clinical trial; ROSC, return of spontaneous circulation; SBP, systolic blood pressure. * Note that SBP rather than MAP was used in this study.

### 2.2. How Can We Achieve Blood Pressure Targets?

Understanding how to raise blood pressure and achieve MAP targets is crucial in AKI management. Mainly, we employ fluids and vasopressors. Guidelines such as the KDIGO Clinical Practice Guideline for Acute Kidney Injury and Surviving Sepsis Campaign recommend administering both fluids and vasopressors in patients with vasomotor shock with, or at increased risk for, AKI [1,7]. However, like with MAP targets, the process has nuances considering factors like the type and dosage of fluids and vasopressors, along with patient comorbidities.

#### 2.2.1. Fluids

Causes of AKI are traditionally placed in three categories: prerenal, intrarenal, and postrenal. Prerenal etiologies, resulting from hypovolemia and kidney hypoperfusion, are often fluid-responsive. Fluids help maintain kidney perfusion and counteract impaired autoregulation. In other contexts fluids may also be helpful, like preoperatively to prevent postoperative acute tubular necrosis (ATN) or with administration of certain medications [22]. Yet, fluids may be detrimental in conditions like cardiogenic shock or obstructive AKI.

##### Fluid Dosing

Determining the appropriate fluid volume for a patient has shifted away from the traditional 30 cc/kg approach, even with the Surviving Sepsis Campaign shifting to “suggesting” rather than “strongly recommending” this strategy in the most recent guidelines [23]. Instead, a more tailored approach to resuscitation is favored, considering the degree of hypovolemia. Excessive fluid resuscitation leads to worse outcomes, including worsening kidney function from potential mechanisms such as intrarenal and abdominal compartment syndrome, venous congestion, and renal oxygen supply-demand mismatch [24,25,26]. 

Recent studies challenge the notion of aggressive fluid resuscitation, even in septic shock. For example, the Conservative versus Liberal Approach to Fluid Therapy of Septic Shock in Intensive Care (CLASSIC) RCT found that among patients with septic shock in the intensive care unit (ICU), intravenous fluid restriction did not result in fewer deaths at 90 days compared to standard intravenous fluid therapy; nor did it impact secondary outcomes like severe AKI [27]. Similar conclusions were drawn from a meta-analysis on sepsis patients, indicating no mortality difference between lower and higher fluid volumes, with some studies even suggesting a higher risk of AKI with excessive fluid [28]. One population commonly seen in the ICU for which this is particularly important is in patients with acute lung injury, where conservative fluid management has been associated with improved lung function without increasing non-pulmonary organ failure [29]. If, as these studies suggest, less fluid is not harmful and could potentially be helpful, we should reconsider intravenous fluid dosing strategies. The dose of fluids administered should be that which is required to maintain perfusion and oxygen delivery to the kidneys and other organs. 

##### Volume Assessment

How can we determine this “sweet spot” in fluid dosing? This largely hinges on a patient’s volume status. Kashani and colleagues outline a comprehensive approach to hemodynamic assessment including history, physical examination, and various invasive and noninvasive monitoring methods [30]. 

History helps identify the type of shock, aiding in categorizing patients as fluid-responsive or not. Physical examination indicators such as dry mucous membranes and delayed capillary refill time suggest hypovolemia or decreased tissue perfusion, respectively, while findings such as lower extremity edema or hepatojugular reflux may indicate non-fluid-responsive etiologies [31,32,33]. Laboratory tests, including serum lactate, offer insights into shock severity and treatment response, though they have limitations in diagnosing etiology [34].

Crude monitoring tools like continuous recording of vital signs, end-tidal carbon dioxide monitoring, urine output measurement, and continuous electrocardiography offer limited sensitivity and specificity in assessing volume status [30]. However, noninvasive methods like passive leg raise can enhance volume assessment by increasing preload, indicating fluid responsiveness. In this maneuver, a patient is positioned with the head of the bed at about 45 degrees, then the bed is adjusted so that the head is horizontal and the patient’s legs are at 45 to 60 degrees, thereby increasing preload by mobilizing blood from the lower extremities to the right heart; increase in cardiac output, stroke volume, or pulse pressure to certain degrees suggests fluid responsiveness [30,35]. Although invasive techniques like thermodilution via pulmonary artery catheter provide direct measurements, noninvasive options such as cardiac output monitors are available. These tools also allow for dynamic assessments of fluid responsiveness, most notably pulse pressure variation (change in pulse pressure during a respiratory cycle, the most studied marker of preload responsiveness) and stroke volume variation (change in stroke volume during a respiratory cycle), with higher values predictive of fluid responsiveness [36]. Administering a fluid challenge is a more direct method to assess fluid responsiveness but it can lead to complications in individuals without hypovolemia.

**Figure 1:**
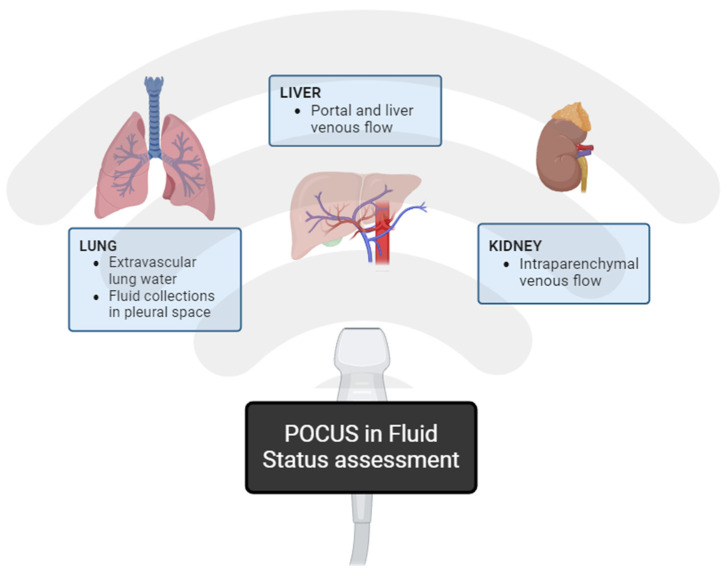
Point of Care Ultrasound (POCUS) in Fluid Status assessment—commonly examined sites.

Point-of-care ultrasound (POCUS) has become a mainstream diagnostic tool for volume assessment, offering insights into hemodynamics and organ congestion [30]. Commonly examined sites include the inferior vena cava (IVC), lung, kidneys, and hepatic vasculature, as outlined in Figure 1. The venous excess ultrasound grading system, or VExUS, combines multiple POCUS findings into a grading system for venous congestion and has been validated in a cardiac surgery population, also noted to be an independent predictor of AKI [37]. However, further validation is needed before widespread adoption due to technical challenges and time constraints [30].

Additionally, invasive tools like arterial lines, pulmonary artery catheters, and central venous lines can aid in volume assessment. These tools can assess responses to passive leg raise and fluid challenges, providing valuable data on a patient’s volume status and guiding therapy, despite variability in outcomes in critically ill patients. [30,38,39,40,41].

##### Choice of Fluid

Fluids can be broken down into crystalloids and colloids, and further within these categories. While colloids have oncotic macromolecules that largely stay in the intravascular space, most crystalloids cause volume expansion via sodium, as they are composed of smaller molecules and electrolytes that lack oncotic properties. This distinction means that colloids remain in the intravascular space longer than crystalloids do and theoretically would suggest that less volume would be required for fluid expansion, but this has not been borne out in a meaningful way in practice [42]. Colloids include gelatins, dextrans, starches, blood, plasma, and perhaps most famously albumin [43]. The most common crystalloids include sodium chloride, lactated Ringer’s, dextrose in water, and Plasma-Lyte. These fluids and their properties are outlined in Table 2.

Multiple studies have explored the optimal fluid for resuscitation, and its impact on kidney function. The Saline versus Albumin Fluid Evaluation (SAFE) study, involving nearly 7000 patients across 16 ICUs, found similar outcomes at 28 days with 4% albumin or normal saline, although albumin showed a higher risk of death in traumatic brain injury cases [49]. A predefined subgroup analysis also found a lower, though not statistically significant, risk of death in the albumin group among patients with severe sepsis. In 2014, the Albumin Italian Outcome Sepsis (ALBIOS) study sought to clarify this and randomly assigned 1818 patients with severe sepsis to either 20% albumin and crystalloid or crystalloid alone, and found no survival benefit at 28 or 90 days, and no difference in AKI [50]. A meta-analysis in patients with sepsis found no difference in risk of requiring KRT between those who received albumin and crystalloids [51]. However, certain colloids like hydroxyethyl starches, as evidenced by The Efficacy of Volume Substitution and Insulin Therapy in Severe Sepsis (VISEP) study, may increase kidney dysfunction and need for KRT in critically ill patients compared to crystalloids [52].

Studies have also compared balanced versus unbalanced crystalloids. Balanced crystalloids contain buffers that mimic plasma composition, while unbalanced fluids like 0.9% sodium chloride lack these buffers [53]. The Isotonic Solutions and Major Adverse Renal Events Trial (SMART) randomized 15,802 adults in five ICUs to receive either normal saline or balanced crystalloids (lactated Ringer’s or Plasma-Lyte A) [54]. It found a lower rate of the composite outcome of death from any cause, new KRT, or persistent kidney dysfunction in the balanced crystalloid group. Individual outcomes did not have significant differences, other than in KRT-free days. The Saline against Lactated Ringer’s or Plasma-Lyte in the Emergency Department (SALT-ED) trial observed similar results in non-critically ill patients [55]. In specific, even though the groups had no difference in hospital-free days, the balanced crystalloid group experienced a lower rate of major adverse kidney events in 30 days. This effect is thought to be driven by the higher chloride concentration of normal saline, which can lead to hyperchloremic metabolic acidosis and reduce renal artery blood flow and GFR [56,57]. A small study of 12 healthy subjects using MRI demonstrated this phenomenon, as infusion of normal saline, but not Plasma-Lyte, led to reduced renal blood flow velocity and renal perfusion compared to baseline [58]. In summary, colloids are not more effective than crystalloids in most settings and sometimes can cause harm, and balanced crystalloids are preferred over unbalanced solutions. However, the choice of fluid for resuscitation depends on volume status, electrolytes, reason for hypovolemia, comorbidities, and many other considerations.

#### 2.2.2. Vasopressors

The other main category of therapy used to increase blood pressure is vasopressors. These are typically recommended when fluid resuscitation fails to meet MAP goals, in some cases of severe hypotension, and for hypotension due to causes other than hypovolemia [23,59,60]. The relationship between fluids and vasopressors is particularly important in AKI in the setting of vasomotor shock, as kidney function can only improve by way of vasopressors once intravascular volume has been restored [61]. 

As with fluids, there are many types of vasopressors. Norepinephrine is the first-choice drug in many cases of shock, with vasopressin as a secondary option per the Surviving Sepsis guidelines [7]. Certain studies suggest vasopressin’s potential superiority in kidney outcomes compared to norepinephrine.

The Vasopressin and Septic Shock Trial (VASST) found no difference in kidney dysfunction or need for KRT between those treated with norepinephrine or vasopressin [62]; however, a post-hoc analysis using the RIFLE criteria found that among patients in the “Risk” category, vasopressin was associated with lower likelihood of progressing to renal “Failure” or “Loss” categories compared to the norepinephrine group, and lower rate of KRT [63]. Similarly, the Vasopressin vs. Norepinephrine as Initial Therapy in Septic Shock (VANISH) RCT showed that early addition of vasopressin to norepinephrine was associated with higher urine volumes and lower rates of KRT in patients with septic shock [64]. Other studies, such as The Vasopressin versus Norepinephrine in Patients with Vasoplegic Shock after Cardiac Surgery (VANCS), terlipressin versus vasopressin infusion in septic shock (TERLIVAP) and smaller studies have also found less kidney injury in the vasopressin groups based on different markers [65,66,67,68]. This suggestion of superior kidney outcomes with vasopressin, particularly compared to norepinephrine, can be explained physiologically. While high-dose norepinephrine can cause decreased kidney and mesenteric blood flow via alpha-1 agonism, vasopressin has been shown to have minimal vasoconstrictive effects on renal afferent arterioles with some vasoconstriction in efferent arterioles, thus improving kidney blood flow [69,70]. Despite potential adverse effects, such as digital ischemia, vasopressin may be preferred in patients with or at risk for AKI [71].

While norepinephrine and vasopressin are frequently studied, other vasopressors like epinephrine and phenylephrine have also been evaluated for their efficacy and safety in different contexts. However, kidney outcomes have either not been reported or shown no difference [72,73]. Notably, a post-hoc analysis of the Angiotensin II for the Treatment of High-Output Shock (ATHOS-3) trial showed that among patients with severe vasodilatory shock on high-dose vasopressors who required KRT, there was faster liberation from KRT when patients received angiotensin II compared to placebo [74,75]. 

Overall, the choice of vasopressor depends on clinical considerations, with vasopressin emerging as a potential renoprotective option. Dosing and combination of these medications are typically determined by MAP goals, as most of these drugs are titratable. The perioperative literature is particularly supportive of “goal-directed therapy” involving hemodynamic targets for fluid resuscitation and inotropic support, with studies demonstrating decreased risk of AKI when such strategies are used [4,76]. One intervention other than fluids and vasopressors that may improve MAP in patients with septic shock is corticosteroid treatment, but the data on other outcomes including mortality and kidney outcomes are limited and inconclusive [77].

## 3. Kidney Replacement Therapy

Ensuring kidney perfusion can help prevent and treat AKI, but inevitably some will require dialysis in the setting of AKI. Here we will explore the indications for KRT and the timing of KRT initiation.

### 3.1. Indications for Dialysis

The most common indications for dialysis have become well known across specialties and levels of training, popularized by the mnemonic AEIOU—acidosis, electrolytes, ingestions, overload (of volume), and uremia. However, initiating dialysis is quite nuanced. For instance, there is the concept of “absolute indications” for dialysis initiation in critically ill patients, with the most common ones listed in Table 3.

For example, some providers will not initiate KRT for a certain blood urea nitrogen (BUN) threshold if there are no uremic complications. Many will attempt to temporize and stave off KRT by first treating reversible causes of these abnormalities. Delaying KRT may be feasible if a patient’s condition remains stable and reversible factors are managed effectively. Table 3 also outlines some “relative” indications for initiation of KRT based on an algorithm by Bagshaw et al., which includes situations in which KRT may be initiated in patients both with and without AKI [78]. 

An additional consideration in KRT initiation, particularly in the critically ill, is dialysis strategy—that is, continuous versus intermittent KRT. While continuous KRT (CKRT) has not shown any conclusive benefit in terms of survival and recovery of kidney function, it allows for slower solute and water removal and thereby is more hemodynamically tolerable, and is thus commonly used when patients in need of KRT are hemodynamically unstable [80]. Other indications for CKRT include patients with cerebral edema or intracranial hypertension and severe electrolyte abnormalities in need of slow correction [81]. One notable population in which intermittent hemodialysis (iHD) is preferred to CKRT is for removal of toxic substances such as salicylates, lithium, and methanol, as these are removed much more effectively and efficiently by iHD [81]

### 3.2. Timing of Dialysis Initiation

Just as there are nuances to *what* should trigger KRT initiation, there are also nuances as to *when*. The landmark clinical trials on the timing of dialysis initiation are outlined in Table 4 and described below. 

The ELAIN study demonstrated improved mortality, shorter hospital stays, and better renal outcomes with early KRT initiation [82]. In contrast, the AKIKI trial, published shortly after, found no mortality benefit but indicated a preference for delayed initiation, as patients in the delayed group spent more time off KRT [83]. However, comparing these studies has been challenged due to ELAIN’s limitations, including its single-center design and specific patient population, post-cardiac surgery patients, which limits generalizability [84]. Additional trials like IDEAL-ICU and STARRT-AKI supported AKIKI’s finding that timing did not affect mortality, with STARRT-AKI also showing benefits in the delayed group [85,86]. AKIKI 2 challenged previous trials by using “delayed” versus “more delayed” groups and found that there indeed may be a limit to how much KRT initiation can be postponed, as the “more delayed” group did experience potential harms [87]. 

Smaller studies have investigated different patient cohorts to provide additional insights into KRT timing. For example, an RCT in western India compared earlier-start (BUN ≥ 70 mg/dL and/or creatinine ≥ 7 mg/dL) versus usual-start dialysis (based on clinical judgment of treating nephrologists) in 208 adults with community-acquired AKI, showing no differences in mortality or dialysis dependence at three months [88]. Although this study included non-critically ill patients, the varying illness severity and lack of blinding posed limitations. 

It is difficult to compare existing studies or to draw meaningful conclusions from them for multiple reasons. First, populations vary widely; even among the main studies in critically ill patients, inclusion criteria range from mild AKI to those with major complications from more severe AKI, and from patients undergoing surgery to those in septic shock. Second, different criteria—namely KDIGO and RIFLE—are used, the latter of which is now out of favor. Third and perhaps most importantly, “early” versus “late” are defined differently in the different studies. Meta-analyses on the subject have similarly struggled to make a strong case for either early or late KRT initiation, but overall support that earlier KRT has no significant survival benefit over later KRT [89].

**Table 4:** Major randomized clinical trials investigating timing of dialysis initiation in acute kidney injury.

Study	Population	Earlier Group	Later Group	Findings	Main Limitations
Effect of Early vs. Delayed Initiation of Renal Replacement Therapy on Mortality in Critically Ill Patients with Acute Kidney Injury (ELAIN), 2016 [82]	231 critically ill patients with KDIGO stage 2 AKI and NGAL > 150 ng/mL	Within 8 h of diagnosis of stage 2 AKI	Within 12 h of developing stage 3 AKI or no initiation	Early KRT initiation was associated with reduced mortality over 90 days, more recovery of renal function, shorter duration of KRT, and hospital stay	One center; almost entirely surgical patients
Artificial Kidney Initiation in Kidney Injury (AKIKI), 2016 [83]	620 critically ill patients with KDIGO stage 3 AKI	Immediately after randomization	If severe hyperkalemia, metabolic acidosis, pulmonary edema, BUN > 112 mg/dL, oliguria for >72 h	Early versus late KRT initiation did not affect mortality, but delayed initiation led to fewer patients on KRT, more KRT-free days, and fewer side effects	Only included patients with stage 3 AKI
Initiation of Dialysis Early Versus Delayed in the Intensive Care Unit (IDEAL-ICU), 2018 [85]	477 patients with early-stage septic shock and RIFLE failure-stage AKI	Within 12 h after documentation of failure-stage AKI	Delay of 48 h after failure-stage AKI if renal recovery had not occurred	Early versus late KRT initiation did not affect overall mortality at 90 days	Used RIFLE criteria; 48 h relatively short time to allow for renal recovery
Standard versus Accelerated Initiation of Renal-Replacement Therapy in Acute Kidney Injury (STARRT-AKI), 2020 [86]	3019 critically ill patients with KDIGO stage 2 or 3 AKI	Within 12 h of developing stage 2–3 AKI (“accelerated”)	If conventional indications developed or AKI persisted for >72 h (“standard”)	Accelerated versus standard KRT initiation did not affect overall mortality at 90 days, but more patients in accelerated group were still on KRT at 90 days and they had more adverse events	Allowed clinicians broad discretion regarding when to initiate KRT in standard group
AKIKI 2, 2021 [87]	278 critically ill patients with KDIGO stage 3 AKI who had oliguria for >72 h or BUN > 112 mg/dL	At time of randomization (“delayed”)	If mandatory indication (noticeable hyperkalemia, metabolic acidosis, or pulmonary edema) developed or BUN reached 140 mg/dL (“more-delayed”)	Longer postponing of KRT initiation did not confer additional benefit and was associated with potential harm, including higher risk of death at 60 days	Used BUN levels as KRT initiation; somewhat different comparison as both groups were somewhat delayed

Green indicates that early KRT showed benefit, blue indicates that late KRT showed benefit, and no color indicates no difference. KDIGO, Kidney Disease Improving Global Outcomes; NGAL, neutrophil gelatinase-associated lipocalin level (plasma); RIFLE, risk, injury, failure, loss, and end-stage kidney disease.

## 4. Medication Considerations

In addition to optimizing blood pressure and evaluating regularly for KRT indications, an important aspect of caring for patients with AKI is medication management. Drug dosing can be challenging in patients with evolving kidney function, but mistakes can lead to complications from underdosing to toxicity, and in some cases can even contribute to worsening AKI.

### 4.1. Factors Affecting Drug Dosing in AKI

Drug dosing in AKI depends on both patient-related and drug-related factors [90]. Patient-related factors include kidney function, electrolyte concentrations, acid-base status, and presence of other organ failures. Dialysis poses additional challenges for drug dosing and brings additional patient factors, such as body weight and volume of distribution, to the forefront.

Kidney function, assessed by GFR, guides dosing recommendations, although estimating GFR can be challenging in critically ill patients. Medication databases and guides offer dosing recommendations based on estimated GFR (eGFR) and creatinine clearance (CrCl) [91]. Typically, drugs requiring “renal dosing” necessitate lower doses with lower GFR. However, estimating GFR in critically ill patients is challenging due to fluctuations in creatinine production and volume of distribution [90]. While incorporating cystatin C into eGFR calculations improves accuracy, this has been demonstrated primarily in outpatient settings, and cystatin C is influenced by factors like inflammation, adiposity, thyroid disorders and medications which can affect the reliability of eGFR calculations [91,92]. 

Electrolyte abnormalities, which are common in AKI, can also affect drug dosing and administration. For example, AKI associated with hyperkalemia may preclude the use of drugs like trimethoprim-sulfamethoxazole or angiotensin receptor blockers. Acid-base status is also relevant since states of metabolic acidosis can reduce response to catecholaminergic drugs and thus require higher doses [93,94,95]. 

Initiating dialysis adds complexities to drug dosing. Clearance of medications changes with dialysis, and dosing must account for both renal and extracorporeal clearance [90]. Continuous versus intermittent dialysis methods require different dosing schemes, as the former allows for constant clearance, while the latter only does so when dialysis is occurring. Furthermore, clearance outside of dialysis sessions relies on patient’s underlying kidney function which is often overlooked. 

Body weight also affects both dialysis and drug dosing and is complicated in the critically ill. Fluid administration and volume depletion can affect a patient’s total body weight, and one must pay close attention to whether drugs should be dosed based on total, ideal, or lean body weight. Hydrophilic medications are typically dosed by total body weight, while hydrophobic medications are dosed based on lean body weight. Additionally, the volume of distribution can increase dramatically in critically ill patients and thus affect the extent to which certain drugs are cleared by dialysis. 

In addition to patient-related factors, a variety of drug-related factors affect drug dosing, especially in patients on dialysis. Critically ill patients often experience low serum albumin levels. Therefore, highly albumin-bound drugs may be found in greater concentrations in their free or unbound forms, increasing risk of toxicity or clearance (and thereby less therapeutic effect) in patients on dialysis [90]. While some have argued that hypoalbuminemia has minimal impact on pharmacologically active drug exposure, this is not the case in patients with impaired clearance mechanisms, like in AKI [96]. 

Hydrophilicity or lipophilicity of a drug is another important factor in drug dosing. Hydrophilic drugs have lower volumes of distribution, are found in the extracellular space, and are predominantly cleared by the kidneys, while lipophilic drugs are found more intracellularly and are cleared more by the liver [90]. Similarly, hydrophilic drugs are more easily removed by KRT and may require dose adjustments based on existing renal function and dialysis dosing. 

Clearance in patients on dialysis is also affected by the molecular weight (MW) of the drug at hand, as those deemed “small molecules” (MW < 500 Dalton (Da)) are removed by diffusion-based dialysis methods, while middle molecules (MW 500–5000 Da) are better removed by convection, and large molecules (>5000 Da) are rarely removed by traditional KRT and require the use of other extracorporeal therapies [90]. There are additional factors that are dependent on dialysis modality, such as the sieving coefficient in convection-based modalities and dialyzer efficacy, that also impact drug dosing. 

### 4.2. Antimicrobial Dosing

Certain classes of medications require extra vigilance in terms of drug dosing, some of the most prominent being antimicrobials, sedatives, antiepileptics, chemotherapy, and diuretics. Here we will focus on antimicrobials, as they are used in many patients with AKI and demonstrate concerns about over- and underdosing.

Overdosing of drugs can lead to specific toxicities, exemplified by cefepime, a commonly used antimicrobial. Cefepime, being hydrophilic and predominantly cleared by the kidneys, can cause neurotoxicity if not appropriately dosed in AKI [97,98]. This can manifest as disorientation, asterixis, hallucinations, and even seizures, and studies have demonstrated significantly higher rates of cefepime-induced neurotoxicity in patients with lower eGFR [99]. These considerations apply to many other common antimicrobials as well, like vancomycin and aminoglycosides, both which have narrow therapeutic indices and require adjustments for renal clearance [100]. Over-dosing vancomycin can be associated with nephrotoxicity, and aminoglycosides with nephrotoxicity, ototoxicity, and neuromuscular blockade [101,102]. To mitigate the potential for such adverse effects, proactive dosing adjustments based on kidney function are crucial for many antimicrobials, but this is not universally done. 

On the other hand, underdosing antimicrobials poses risks of inadequate treatment and increased resistance, particularly in patients undergoing CKRT. As an example, cefepime’s hydrophilic nature allows it to be removed by CKRT, and other features of the drug such as its low protein binding affinity, enhance this susceptibility [103,104,105]. Without considering drug and patient factors, antimicrobial levels may not reach minimum inhibitory concentration (MIC), fostering bacterial growth and antimicrobial resistance. While the efficacy of beta-lactam antibiotics like cefepime is mostly time-dependent, aminoglycosides and vancomycin are dependent on concentration (and vancomycin on both), requiring strategies such as loading doses and/or extended dosing intervals to achieve target doses and minimize complications related to under-dosing [100]. Similar concerns apply to other drug classes, emphasizing the importance of careful dosing based on individual factors to ensure both safety and efficacy [106,107].

While the resistance issue is more specific to antimicrobials, the same principles apply to other drug classes—for example, antiepileptic overdosing can lead to toxicities like oversedation, gastrointestinal distress, and altered mental status, depending on the drug at hand, and underdosing can lead to breakthrough seizures. Careful attention to patient and drug-related factors is essential to optimize both drug safety and drug efficacy.

## 5. Discussion

Blood pressure optimization, appropriate use of KRT, and medication management stand as key objectives in AKI management, though controversies persist. Various treatments, including *N*-acetylcysteine, atrial natriuretic peptide, statins, insulin growth factor, and a variety of other agents have been explored but often disproven [108]. Emerging options like alkaline phosphatase and L-Carnitine in sepsis-associated AKI, vitamin D in hospitalized AKI, and p53-targeted short interfering RNA in post-cardiac surgery AKI are under investigation, alongside non- pharmacologic interventions such as extracorporeal devices and remote ischemic preconditioning. Nephrotoxic agents, such as non-steroidal anti-inflammatory drugs, aminoglycosides, certain chemotherapeutic agents, and dozens of others, should be avoided as best as able in AKI, but this is more to minimize harm than to achieve unique benefits [109]. 

Oftentimes, the most important “treatments” for AKI are, rather, addressing complications of the AKI rather than the AKI itself. Most of these interventions are also debated. Some of the most controversial are diuretics and bicarbonate. Regarding diuretics, KDIGO recommends not using diuretics to treat AKI except for cases of volume overload but not in cases of oliguria without volume overload, for example [1]. Some research has supported this, such as the Fluids and Catheters Treatment Trial (FACTT) [29]. This study showed that higher furosemide doses in patients with acute lung injury were associated with decreased mortality at 60 days, but this became nonsignificant after adjusting for post-AKI fluid balance, indicating that perhaps the benefit was related more to volume than the diuretic itself. Furthermore, a meta-analysis on the subject found that the use of diuretics in AKI was not significantly associated with mortality risk or KRT requirement [110]. As for bicarbonate supplementation, there is very limited data from RCTs, making it particularly contentious [111]. Perhaps the best-known trial, Sodium bicarbonate therapy for patients with severe metabolic acidaemia in the intensive care unit (BICAR-ICU), found that administration of sodium bicarbonate to patients with severe metabolic acidemia did not affect the composite outcome of death by day 28 or presence of at least one organ failure at day 7, but did decrease the outcome and day 28 mortality in patients with AKI [112]. Other common complications and potential treatments are presented in Table 5. 

## 6. Future Directions

In addition to completing the trials on potential AKI treatments that are underway, there are other avenues for improving AKI management. Most of the existing data on volume resuscitation, fluid status, and blood pressure optimization are in critically ill patients and/or peri-surgical patients. This leaves large populations under-studied or unstudied, including hospitalized non-critically ill patients and adults in the community with AKI. While AKI is much more common proportionally in the ICU, community-acquired AKI is the most common form of AKI worldwide, and attention to epidemiology and management of AKI in such populations thus has the potential to reduce overall burden of AKI [117,118]. Tools to promote early recognition of AKI—such as electronic alerts—are also being implemented, and with appropriate training may allow for earlier interventions like medication adjustments that can minimize complications [119]. Continued identification of novel biomarkers beyond serum creatinine to identify and adjudicate AKI may also contribute to earlier recognition and more targeted interventions for AKI. Similarly, specific therapies that benefit kidney regeneration, such as stem cells, should continue to be investigated [120]. 

## 7. Conclusions

AKI is highly prevalent and is associated with significant morbidity and mortality. Therefore, recognition, prevention, and treatment of its complications is of utmost importance. Here we presented three cornerstones of AKI management: blood pressure optimization, effective use of KRT, and medication management. However, even these principles exist in a background of controversy and evolving data. Targeted therapies for AKI and management of AKI complications are being researched, but as of yet no type of AKI has a “magic bullet” therapy that can reliably restore kidney function. Continued refinement of the existing data, as well as exploration into novel therapies and interventions, are essential in reducing the incidence and complications of AKI.

## Figures and Tables

**Table 2:** Types and properties of most commonly used fluids.

Fluid	NS	LR	Plasma-Lyte A	D5W	5% Albumin	25% Albumin	Blood
Composition							
Na^+^ (mEq/L)	154	130	140	0	154	154	135–145
Cl^−^ (mEq/L)	154	109	98	0	154	154	94–111
K^+^ (mEq/L)	0	4	5	0	0	0	4.5–5
Ca^2+^ (mEq/L)	0	3	0	0	0	0	2.2–2.6
Mg^2+^ (mEq/L)	0	0	3	0	0	0	0.8–1
Lactate (mEq/L)	0	28	0	0	0	0	1–2
Gluconate (mEq/L)	0	0	23	0	0	0	0
Acetate (mEq/L)	0	0	27	0	0	0	0
Glucose (g/L)	0	0	0	50	0	0	70–120
Albumin (g/L)	0	0	0	0	50	250	0
**Osmolarity (mOsm/L)**	308	275	294	278	308	308	275–290
**pH**	5.4	6.5	7.4	4.2	7.4	7.4	7.4
**Distribution (mL per 1 L infusion)**							
Intracellular	0	0	0	667	0	0	0
Interstitial	750	750	750	250	100	100	0
Intravascular	250	250	250	83	900	900	1000

D5W, dextrose 5% in water; LR, lactated Ringer’s; NS, normal saline. Adapted from [44,45,46,47,48].

**Table 3:** Indications for initiation of kidney replacement therapy.

**“Absolute” Indications**	
**Indication**	**Associated Characteristic(s)**
**Azotemia**	BUN ≥ 100 mg/dL
**Uremic complications**	Encephalopathy Pericarditis Bleeding
**Hyperkalemia**	K ≥ 6 mEq/L ECG abnormalities
**Hypermagnesemia**	Mg ≥ 8 mEq/L Anuria Absent deep tendon reflexes
**Acidosis**	Serum pH ≤ 7.15
**Oligo-anuria**	Urine output < 200 mL in 12 h Anuria
**Fluid overload**	Diuretic-resistant pulmonary edema in presence of AKI
**Ingestion**	Depends on ingested substance and antidotes available
**“Relative” Indications**	
**In setting of AKI**	Severe AKI Severely progressive AKI Severe illness Poor trajectory Poor response to resuscitation
**In absence of AKI**	Acute liver failure Dysthermia Refractory septic shock Severe TLS Severe electrolyte disturbances (e.g., dysnatremia)

AKI, acute kidney injury; BUN, blood urea nitrogen; ECG, electrocardiogram; K, potassium; KRT, kidney replacement therapy; TLS, tumor lysis syndrome. Adapted from [78,79].

**Table 5:** Common complications of AKI and therapeutic options.

Complications	Therapeutic Options
**Azotemia**	Lower protein load through diet Avoid medications that can increase BUN when feasible (e.g., corticosteroids)
**Hyponatremia**	Limit water intake
**Hyperkalemia**	Shifting therapies: Beta-agonists (e.g., albuterol), insulin + glucose Potassium resin exchangers: sodium zirconium cyclosilicate (Lokelma) or patiromer (Veltassa) Note: Ensure an intact colon for these therapies [113] Dialysis
**Hyperphosphatemia**	Use of phosphate binders is controversial; caution in AKI patients on dialysis, especially with CKRT [114,115] Note: Binders may not be suitable for patients not eating
**Metabolic Acidosis**	Consider alkali therapy; indications vary and are controversial, although a survival benefit in AKI has been described [112,116] Options include sodium bicarbonate tablets, sodium citrate/citric acid (Bicitra), IV sodium bicarbonate
**Volume overload**	Diuretics Dialysis

CKRT, Continuous Kidney Replacement Therapy.
